# Supplementary material for: Conscientious Objection and Other Motivations for Refusal to Treat in Hastened Death: A Systematic Review
Source: Healthcare (Basel). 2023 Jul 26;11(15):2127. doi: 10.3390/healthcare11152127 (PMC10418655; doi:10.3390/healthcare11152127)
Supplement: Supplementary file 1 [file healthcare-11-02127-s001.zip › healthcare-2502169-supplementary.pdf]

Supplementary Materials

Supplement material 1 – Search Strategy of the systematic review

Table S1 - Search strategy of the systematic review for each database

Data base	Search strategy
**PubMed**	(refusal to treat[MeSH Terms])) OR (physician refusal to treat[MeSH Terms]) OR (conscientious objection[Text Word])<br>AND<br>(euthanasia[MeSH Terms]) OR (assisted suicide[MeSH Terms])) OR (assisted suicides[MeSH Terms])<br><br>Search Field: Mesh terms and Text Words<br>Filters: No filters were used
**Scopus**	“refusal to treat” OR “physician refusal to treat” OR “conscientious objection”<br>AND<br>“euthanasia” OR “assisted suicide” OR “assisted suicides”<br><br>Search Field: Topic<br>Filters: No filters were used
**Web of Science**	“refusal to treat” OR “physician refusal to treat” OR “conscientious objection”<br>AND<br>“euthanasia” OR “assisted suicide” OR “assisted suicides”<br><br>Search Field: Article title, Abstract, Keywords<br>Filters: No filters were used

Supplementary Material 2 - Let Evidence Guide Every New Decision (LEGEND) tool[1]

Table S2. Quality analysis of the qualitative studies included in the systematic review

	Bouthillier, 2019 [2]	Isaac, 2019 [3]	Brown, 2021 [4]	Dumont, 2019 [5]	Haining, 2021 [6]	Beuthin, 2018 [7]	Pesut, 2020 [8]	Brown, 2021 [9]
Do the study purpose/objectives and inclusion/exclusion criteria assist in answering your clinical question?	Yes	Yes	Yes	Yes	Yes	Yes	Yes	Yes
Is a qualitative study congruent with the author's study purpose above?	Yes	Yes	Yes	Yes	Yes	Yes	Yes	Yes
General Questions								
1- Was a qualitative design clearly identified?<br>• What was the qualitative design?	Yes<br>• Other, exploratory qualitative study	Yes<br>• Grounded Theory	Yes<br>• Other, descriptive qualitative study	Yes<br>• Other, descriptive qualitative study	Yes<br>• Phenomenology	Yes<br>• Narrative	Yes<br>• Other, descriptive qualitative study	Yes<br>• Other, descriptive qualitative study
2- Is the area of study clearly stated in one sentence?	Yes	Yes	Yes	Yes	Yes	Yes	Yes	Yes
3- Was the design appropriate to explore the area of study being studied?	Yes	Yes	Yes	Yes	Yes	Yes	Yes	Yes
4- Was the guiding framework identified?	Yes	Yes	Yes	Yes	Yes	No	Yes	Yes
5- Was the guiding framework appropriate for the area of study being evaluated?	Yes	Yes	Yes	Yes	Yes	Unknown	Yes	Yes
6- Was the setting clearly identified for the area of study being studied?<br>• Was the setting appropriate for the area of study being studied?	Yes	Yes	Yes	Yes	Yes	Yes	Yes	Yes
7- Was the context of the participants analysed using the words of the participants?	Yes	Yes	Yes	Yes	Yes	Yes	Yes	Yes
CREDIBILITY: Are the findings credible?								

8- Was the researcher known and trusted by the participants?<br>• How was trust developed among the participants?<br>• How long was the researcher in the environment of the participants prior to collecting data?	Unknown	No	Unknown	Unknown	Unknown	Unknown	Unknown	Unknown
CONFIRMABILITY: Are the findings verified within the context?								
9- Did the researcher report how findings (themes) were confirmed?<br>• How were themes confirmed?	Yes<br>• Experts	Yes<br>• Participants	Yes<br>• Participants	Unknown	Yes<br>• Participants	Unknown	Yes<br>• Use of Field Notes	Yes<br>• Participants
MEANING IN CONTEXT: Are the findings reported within the context of the area of study?								
10- Does the researcher discuss the findings (themes) within a socio-cultural context?<br>• Were the findings reported in terms of the context:	Yes<br>• of the participants	Yes<br>• of the participants	Yes<br>• of the participants	Yes<br>• of the participants	Yes<br>• of the participants	Yes<br>• of the participants	Yes<br>• of the participants	Yes<br>• of the participants
SATURATION: Was the data collected until there was no new information coming forth?								
11- Was saturation of data discussed and reached?<br>• If yes, what was the sample size (number of participants)?<br>• If no, were there at least 10 participants (N>10)?<br>• Was the sample size justified in the discussion?	No<br>• Yes, 22 participants	Yes<br>• n=40	No<br>• Yes, 35 participants	Yes<br>• n=20	Yes<br>• n=17	No<br>• Yes, 17 participants	No<br>• Yes, 59 participants	No<br>• Yes, 35 participants
RECURRENT PATTERNING: Is there consistency in repeated patterns, themes, & acts over time?								
12- Was the data analysis method identified?	Yes	Yes	Yes	Yes	Yes	Yes	Yes	Yes
13- Were the themes reported in terms of the theoretical framework?<br>• Were the themes supported by raw data?<br>• Did the raw data fall into patterns?<br>• Were patterns reported as themes?	Yes	Yes	Yes	Yes	Yes	Yes	Yes	Yes
TRANSFERABILITY: Are the findings transferable?								
14- Is this information gained from the study applicable to similar groups and contexts?<br>• What is applicable to my patient population?	Yes	Yes	Yes	Yes	Yes	Yes	Yes	Yes
QUALITY LEVEL / EVIDENCE LEVEL	Good Quality Qualitative Study	Good Quality Qualitative Study	Good Quality Qualitative Study	Good Quality Qualitative Study	Good Quality Qualitative Study	Good Quality Qualitative Study	Good Quality Qualitative Study	Good Quality Qualitative Study

Table S3. Quality analysis of the quantitative studies included in the systematic review

	Velasco, 2022 [10]	Nordstrand, 2014 [11]
Do the study aim/ purpose/ objectives and inclusion/exclusion criteria assist in answering your clinical question?<br>• Study Aim/ Purpose/ Objectives<br>• Inclusion Criteria<br>• Exclusion Criteria	Yes<br>Objective: To identify the opinion and attitudes of nurses in the Community of Madrid on the regulation of Euthanasia and Medically assisted suicide in Spain.<br>Inclusion criteria: Members of the Official College of Nurses of Madrid.	Yes<br>Objective: To examine medical students' views on conscientious objection and controversial medical decisions.<br>Inclusion criteria: Not referred
Is a cross-sectional study congruent with the author's study aim/ purpose/ objectives above?	Yes	Yes
VALIDITY: Are the results of the cross–sectional study valid or credible?		
1- Are the study methods clearly described and appropriate for the question?<br>• Is the setting clearly described and appropriate?<br>• Was there a representative sample of patients at a well-defined point in the course of the condition of interest?<br>• Is the sample population clearly described and sufficient?	Yes	Yes
2- Were the participants similar (homogeneous) with respect to known factors of interest (e.g., demographic, exposure, risk, treatment, or etiology)?	Yes	Yes
3- Were objective and unbiased criteria used to measure the variable of interest?<br>• Was the variable of interest quantifiable and precisely measurable?<br>• Were instruments used to measure the variable of interest tested to be valid and reliable?	Yes	Yes
4- Was there freedom from conflict of interest?<br>• Sponsor/Funding Agency or Investigators	Yes	Yes
RELIABILITY: Are these valid study results important?		
5- Did the study have a sufficiently large sample size?<br>• Was a power analysis described?<br>• Did the sample size achieve or exceed that resulting from the power analysis?<br>• Did each subgroup also have sufficient sample size (e.g., at least 6 to 12 participants)?	Yes	Yes
6- Were the statistical analysis methods appropriate?<br>• Were the statistical analysis methods clearly described?<br>• If subgroups were evaluated, was a statistical adjustment made for the differences?	Yes	Yes

7- What are the main results of the study? (e.g., Helpful data: Page #, Table #, Figures, Graphs)<br>• For a Prevalence Study: What is the rate (e.g., number per population)?<br>• For an Etiology Study: How strong is the association/correlation between known factors and the variable of interest?<br>• What were the measures of statistical uncertainty (e.g., precision)? (Were the results presented with Confidence Intervals or Standard Deviations?)	Conscientious objection being related with age, years of work experience, model of care and above all religious beliefs. Table 4, Figure 3 and 4.	Moral and religious reasons for conscientious objection were identified. The impact of religion was also discussed. Table 4.
8- . Were the results statistically significant?	Yes	Yes
9- Were the results clinically significant?<br>• If potential confounders were identified, were they discussed in relationship to the results?	Yes	Yes
APPLICABILITY: Can I apply these valid, important study results to my population?		
10- Can the results be applied to my population of interest?<br>• Is the setting of the study applicable to my population of interest?<br>• Were the participants in this study similar to my population of interest?<br>• Does the variable of interest apply to my population or question of interest?	Yes	Yes
11- Are my patient's and family's values and preferences satisfied by the knowledge gained from this study?	Yes	Yes
12- Would you include this study/article in development of a care recommendation?	Yes	Yes
QUALITY LEVEL / EVIDENCE LEVEL	Good Quality Cross-Sectional Study	Good Quality Cross-Sectional Study

Table S4. Quality analysis of the expert opinion/review studies included in the systematic review

	Pesut, 2019 [12]	Carpenter, 2020 [13]
Do the aim/purpose/objectives assist in answering your clinical question?	Yes	Yes
Basic Elements of an Expert Opinion/Review Article		
1- Is the author a known expert in the field being studied?	Yes	Yes
2- Does the author have a known bias?	No	No
3- Is the patient population, problem, or issue clearly described?	Yes	Yes
4- Is the literature search clearly described?	No	No
5- Is the date range of the cited literature appropriate and current?	Yes	Yes
6- What types of research are cited (e.g., animal model, basic science, clinical studies)?	Basic science and clinical studies	Basic science and clinical studies
7- Is more than one point of view explained, reported, or referenced?	Yes	Yes
8- Were any conclusions clearly presented in the article?<br>• If applicable, were any adverse events clearly described?	Yes	Yes
9- Was there freedom from conflict of interest?<br>• Sponsor/Funding Agency or Authors	Unknown	No
Applicability: Can I Apply this Expert Opinion / General Review Information?		
10- Can the results be applied to my population of interest?<br>• Is the setting described in the article applicable to my population of interest?<br>• Do the patient outcomes apply to my population or question of interest?<br>• Were the patients in this article similar to my population of interest?	Yes	Yes
11- Are my patient's and family's values and preferences satisfied by the knowledge gained from this article (such as outcomes considered)?	Yes	Yes
12- Would you include this article in development of a care recommendation?	Yes	Yes
Quality Level / Evidence Level	Good Quality Expert Opinion / General Review	Good Quality Expert Opinion / General Review

Supplementary Materials

[1] Clark E, Burkett K, Stanko-Lopp D. Let Evidence Guide Every New Decision (LEGEND): An evidence evaluation system for point-of-care clinicians and guideline development teams. *J Eval Clin Pract*. 2009;15(6):1054-1060. doi:10.1111/j.1365-2753.2009.01314.x

[2] Bouthillier M-E, Opatrny L. A qualitative study of physicians' conscientious objections to medical aid in dying. *Palliat Med*. 2019;33(9):1212-1220. doi:10.1177/0269216319861921

[3] Isaac S, Mclachlan A, Chaar B. Australian pharmacists' perspectives on physician-assisted suicide (PAS): thematic analysis of semistructured interviews. *BMJ Open*. 2019;9(10). doi:10.1136/bmjopen-2018-028868

[4] Brown J, Goodridge D, Thorpe L, Crizzle A. "What Is Right for Me, Is Not Necessarily Right for You": The Endogenous Factors Influencing Nonparticipation in Medical Assistance in Dying. *Qual Health Res*. 2021;31(10):1786-1800. doi:10.1177/10497323211008843

[5] Dumont I, Maclure J. Conscientious objection to medical assistance in dying: A qualitative study with Quebec physicians [Objection de conscience et aide médicale à mourir: Une étude qualitative auprès de médecins Québécois]. *Can J Bioeth*. 2019;2(2):110-134. doi:10.7202/1065691AR

[6] Haining CM, Keogh LA, Gillam LH. Understanding the Reasons Behind Healthcare Providers' Conscientious Objection to Voluntary Assisted Dying in Victoria, Australia. *J Bioeth Inq*. 2021;18(2):277-289. doi:10.1007/s11673-021-10096-1

[7] Beuthin R, Bruce A, Scaia M. Medical assistance in dying (MAiD): Canadian nurses' experiences. *Nurs Forum*. 2018;53(4):511-520. doi:10.1111/nuf.12280

[8] Pesut B, Thorne S, Storch J, Chambaere K, Greig M, Burgess M. Riding an elephant: A qualitative study of nurses' moral journeys in the context of Medical Assistance in Dying (MAiD). *J Clin Nurs*. 2020;29(19-20):3870-3881. doi:10.1111/jocn.15427

[9] Brown J, Goodridge D, Thorpe L, Crizzle A. "I Am Okay With It, But I Am Not Going to Do It": The Exogenous Factors Influencing Non-Participation in Medical Assistance in Dying. *Qual Health Res*. 2021;31(12):2274-2289.

doi:10.1177/10497323211027130

[10] Velasco Sanz TR, Cabrejas Casero AM, Rodríguez González Y, Barbado Albaladejo JA, Mower Hanlon LF, Guerra Llamas MI. Opinions of nurses regarding Euthanasia and Medically Assisted Suicide. *Nurs Ethics*. Published online 2022. doi:10.1177/09697330221109940

[11] Nordstrand SJ, Nordstrand MA, Nortvedt P, Magelssen M. Medical students' attitudes towards conscientious objection: a survey. *J Med Ethics*. 2014;40(9):609-612. doi:10.1136/medethics-2013-101482

[12] Pesut B, Thorne S, Greig M. Shades of gray: Conscientious objection in medical assistance in dying. *Nurs Inq*. 2019;27(1). doi:10.1111/nin.12308

[13] Carpenter T, Vivas L. Ethical arguments against coercing provider participation in MAiD (medical assistance in dying) in Ontario, Canada. *BMC Med Ethics*. 2020;21(1). doi:10.1186/s12910-020-00486-2
